# Psychoeducation Reduces Postoperative Analgesic Consumption and Mobilization Period After Spine Surgery: A Controlled Clinical Trial

**DOI:** 10.3390/brainsci16020179

**Published:** 2026-01-31

**Authors:** Judit Sütő, Álmos Klekner, Andor Karácsony, János Nagy, Andrea Bakó, Anita Szemán-Nagy, József Virga

**Affiliations:** 1 Department of Oncology, Faculty of Medicine, University of Debrecen, H-4032 Debrecen, Hungary; 2 Department of Neurosurgery, Faculty of Medicine, University of Debrecen, H-4032 Debrecen, Hungary; aklekner@gmail.com (Á.K.);; 3 Faculty of Humanities, Institute of Psychology, University of Debrecen, H-4032 Debrecen, Hungary

**Keywords:** patient education as topic, anxiety, preoperative care, postoperative pain management

## Abstract

**Background**: Spine surgeries present challenges for patients, including postoperative pain and difficulties with mobilization. Studies indicate that fear and anxiety prolong recovery; multidisciplinary care, including psychoeducation, which informs patients about their condition, addresses emotional challenges, and teaches coping strategies have benefits on recovery. **Objectives**: This study investigated whether preoperative psychoeducation improves outcomes in spinal surgery by reducing postoperative analgesic use and accelerating mobilization, with the hypothesis that it decreases medication needs and shortens recovery time. **Methods**: Data of 100 patients operated on spinal disease were analysed: 50 of them underwent microscope-assisted discectomy for lumbar disc herniation (LDH), and 50 were treated with transpedicular posterior lumbar interbody fusion (PLIF) for monosegmental instability. Each group was subdivided into a psychoeducation group (N = 25) and a control group (N = 25). All patients completed the Surgical Fear Questionnaire (SFQ). Postoperative analgesic use and time to mobilization were analysed. **Results**: Patients receiving psychoeducation in both groups reported lower preoperative anxiety, required fewer analgesics, and, in the PLIF group, achieved earlier mobilization. A strong correlation was found between SFQ scores and analgesic consumption (*p* < 0.01). **Discussion**: Preoperative psychoeducation reduced anxiety, decreased postoperative analgesic use, and enhanced mobilization, suggesting clinical and economic benefits if integrated into standard care.

## 1. Introduction

To enhance postoperative recovery, recent clinical guidelines increasingly recommend multidisciplinary perioperative care and patient support [1,2,3]. Within this framework, psychoeducation is recognized as a structured method of information delivery aimed at increasing patients’ understanding of their illness, supporting emotional processing, and teaching effective coping strategies [4].

A growing body of interdisciplinary research has shown that preoperative fear and anxiety significantly influence postoperative outcomes [5,6]. For example, one study examined the impact of a brief group psychoeducational intervention on patients undergoing coronary artery bypass grafting (CABG), finding that patients receiving psychoeducation reported lower fear scores compared to those receiving routine care [7]. Similarly, the use of audiotaped educational programs in CABG patients was associated with reduced levels of postoperative anxiety and depression [8]. A systematic review analyzing four different psychoeducational programs—each involving education about anxiety and training in anxiety-reduction techniques—concluded that such interventions can be effective in reducing preoperative anxiety [9].

Spinal decompression and fusion surgeries are increasingly common, while healthcare systems face growing financial constraints [10]. In 2017, over five million spine surgeries were performed worldwide, making spinal surgery one of the fastest-growing surgical fields [11]. Numerous studies have examined the economic impact of these procedures, particularly in the treatment of degenerative lumbar spine conditions such as lumbar disc herniation (LDH) and posterior lumbar interbody fusion (PLIF). These analyses have included cost evaluations, cost-effectiveness assessments, and incremental cost-effectiveness ratios to identify ways to optimize treatment protocols and reduce overall healthcare expenditures [12,13]. The impact of different surgical techniques on perioperative parameters—such as length of hospital stay—has also been widely studied [14]. Several systematic and critical reviews have evaluated the cost-effectiveness of various surgical approaches to cervical and lumbar spine disorders [15].

Prior interdisciplinary research has demonstrated that preoperative anxiety can adversely affect surgical outcomes and that psychoeducational interventions may moderately reduce postoperative pain and promote earlier mobilization across different patient groups [7,16,17,18]. These findings support the hypothesis that postoperative analgesic use and mobilization are associated with patients’ preoperative anxiety levels.

This study investigates whether preoperative psychoeducation can reduce the need for analgesic medication and shorten the mobilization period following spinal surgery. We hypothesize that patients who receive psychoeducation prior to surgery will demonstrate reduced or eliminated analgesic use and faster postoperative mobilization, particularly after PLIF procedures.

## 2. Materials and Methods

### 2.1. Study Design

This study was designed as a quasi-randomized controlled trial (RCT) with a parallel-group design and single-blind methodology. The study group received preoperative psychoeducation prior to spine surgery, while the control group received standard care without psychoeducation. The allocation ratio was 1:1 (study group: control group). Patient selection for the study was made at the Department of Neurosurgery, University of Debrecen, between 2021 and 2023.

Randomization was implemented by allocating incoming patients to the study groups in an alternating manner, independently of all other parameters. This approach was intended to minimize selection bias. In this sense, randomization was assumed to be ensured by the fact that patients presented to the clinic in a completely unscreened and inherently random order, thereby approximating a form of spontaneous randomization. Outcome assessors, nursing staff, and data analysts were all blinded to group allocation.

### 2.2. Patients

The study and control groups were composed of randomly selected patients, aged 35 to 75 years, who had been referred from the neurosurgical outpatient clinic with an indication for single-level lumbar disc herniation or spinal instability surgery. The age range was established by the neurosurgeon to ensure homogeneity across the groups and to minimize the potential benefit of early recovery in younger patients. To further ensure group homogeneity, inclusion criteria required patients to have a body mass index (BMI) of less than 40. Exclusion criteria included severe comorbidities that could affect the need for analgesics or postoperative mobilization, such as diabetes, rheumatoid arthritis, malignant diseases, previous spinal surgery, Parkinson’s disease, a history of stroke with reduced physical activity or disability (Karnofsky performance status below 70), inability to collaborate with a psychologist, use of psychiatric medications, or a BMI over 40. All participants were briefed on the study objectives and signed an informed consent form. The study adhered to the principles outlined in the Declaration of Helsinki and was approved by the Ethical Committee of the University of Debrecen, Hungary.

### 2.3. Statistical Analysis

To compare analgesic consumption and mobilization duration between the psychoeducation and control groups, the Mann–Whitney U test was used, with a significance level set at *p* ≤ 0.01. The correlation between preoperative anxiety scores and postoperative drug consumption was assessed using Spearman’s rank correlation test.

### 2.4. Psychoeducation

Psychoeducation was conducted by a licensed psychologist through private, one-on-one sessions with each patient in the study group prior to surgery. The psychoeducational protocol was developed by the authors, based on the cited references [9]. All patients received the same basic information. The primary focus was on reducing information deficits and alleviating fear of the unknown. Key components included providing information about pre- and postoperative events, ensuring familiarity with the clinical environment, describing conditions that respect patient privacy and dignity, offering a hospital introduction illustrated with photo documentation, presenting the operating theater block, outlining postoperative medication provision, and explaining the process of postoperative mobilization.

Session duration depended on the individual needs of the patient and was not strictly predefined; instead, it was adapted to the patient’s coping style and psychological preparedness. The average length of the sessions was 35 min +− 17 min. Nevertheless, all patients received a single psychoeducational session in accordance with the standardized protocol described above. The psychoeducational protocol was divided into two main components: preoperative instructions, which covered tasks and preparations for the day prior to surgery, and postoperative guidance, which focused on recovery and care following surgery. The scope and depth of the information were individualized, taking into account each patient’s coping mechanisms and psychological preparedness. The psychoeducational session addressed anticipated preoperative responsibilities, such as the timing of the last oral intake before surgery.

Patients were informed about the administration of a mild anxiolytic drug to help alleviate preoperative anxiety. the transport to the operating theatre, the opportunity to bid farewell to accompanying family members, the methods of general anesthesia. Details of postoperative events are also described, the transfer back to their bed in the ward and use of routine postoperative intravenous analgesia. Mobilization is not allowed until the following day, and patients use a bedpan with nursing assistance until then. On postoperative day one, patients are first mobilized with assistance from nursing staff, and if their condition allows, they are helped to change into their own clothes. A physiotherapist visits each patient the day after surgery to provide personalized instructions for relevant postoperative exercises. Patients are also educated on the use of the nurse call system, located above each bed. They are advised to use this system to promptly report symptoms such as pain or nausea, which would be addressed immediately by the nursing staff. Contact information for the ward’s nursing station is provided to each patient and can be shared with family members to facilitate communication about the patient’s condition at any point during the perioperative period.

To further reduce anxiety and unfamiliarity, patients are shown visual aids depicting the inpatient ward, operating theatre, waiting area, and nurses’ station. This approach aims to familiarize patients with the care environment and reduce procedural anxiety.

Each patient in the study group received psychoeducation, after which all patients in both the study and control groups completed the validated Hungarian version of the Surgical Fear Questionnaire (SFQ) (Supplementary Materials) prior to surgery. This questionnaire has been validated and widely used for assessing surgical patients’ fear of surgery [19,20]. The SFQ is a reliable 10-item instrument that measures surgical fear across two subscales: fear of short-term consequences of surgery and fear of long-term consequences [21].

### 2.5. Evaluation of Postoperative Analgesic Consumption and Mobilization Period

As the focus of the study was on patients’ postoperative analgesic requirements, rather than on the subjective, individual-dependent intensity of pain perception, the assessment was directed toward the objective manifestation of pain in terms of medication consumption; therefore, pain intensity scales were not applied. Furthermore, due to the limited range and quantities of analgesic medications used, a non-standard analgesic consumption score was employed. Analgesic scores represent the number of daily requests for additional pain medication, summed over the entire duration of the patient’s hospital stay.

The day after surgery, patients’ analgesic consumption was assessed based on a two-step protocol established by the department: if the patient complained of pain, the first intervention was an intravenous infusion of diclofenac (75 mg). If pain persisted, 1 g of metamizole sodium was administered intravenously, with the option for repeated doses as necessary. Analgesic use was recorded daily and categorized as follows: a score of 0 was assigned if no additional analgesics were needed beyond the prescribed routine for that day; a score of 1 was given if the patient received only metamizole sodium; a score of 2 was given if diclofenac infusion alone was administered; and a score of 3 was assigned if both diclofenac and metamizole sodium were used. This process was repeated and recorded daily throughout the patient’s hospital stay.

Mobilization was measured by the time it took for the patient to first get out of bed unassisted and take a few steps. All mobilization was supervised by a physiotherapist. For statistical analysis, the number of days post-surgery when the patient first mobilized independently was recorded.

## 3. Results

**Figure 1:**
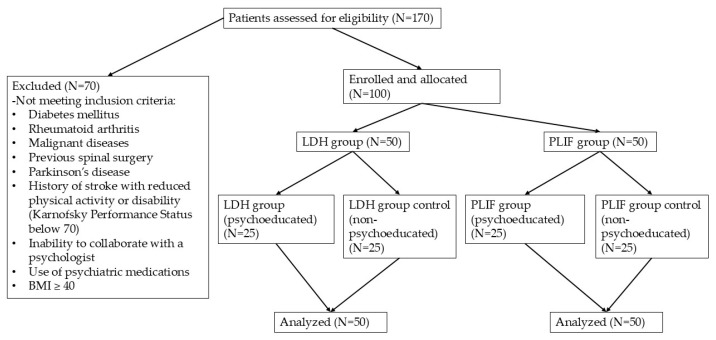
Flowchart illustrating the enrollment, allocation, and analysis of patients in the study.

Data from 50 patients who underwent lumbar disc herniation (LDH) surgery (monosegmental microscope-assisted herniotomy-discectomy) and 50 patients who underwent spinal fusion surgery (posterior lumbar interbody fusion [PLIF]) for monosegmental instability at the Department of Neurosurgery, University of Debrecen, Hungary, were analyzed (Table 1). All patients within each group underwent the same neurosurgical procedure, supervised by the same senior neurosurgeon: unilateral dorsal microdiscectomy for the LDH group (N = 50) and monosegmental decompression and fusion with transpedicular screws and intervertebral cage for the PLIF group (N = 50). In each group, 25 patients received preoperative psychoeducation (study group) and 25 did not (control group). There was found no significant differences in the clinical data between the study and control groups (Figure 1, Table 1).

Out of 170 patients assessed for eligibility, 70 were excluded based on predefined exclusion criteria (e.g., comorbidities, inability to collaborate, BMI ≥ 40). The remaining 100 patients were enrolled and allocated into two surgical groups: lumbar disc herniation (LDH) and posterior lumbar interbody fusion (PLIF), each comprising 50 patients. Within each group, patients were further divided into psychoeducated and non-psychoeducated (control) subgroups (N = 25 per subgroup). No participants dropped out, and all enrolled patients were included in the final analysis.

### 3.1. Preoperative Anxiety in the LDH Group

The “Surgical Fear Questionnaire” (SFQ) was completed by all patients in both the psychoeducational (LDH study group) and non-psychoeducational (LDH control group) groups prior to surgery.

**Figure 2:**
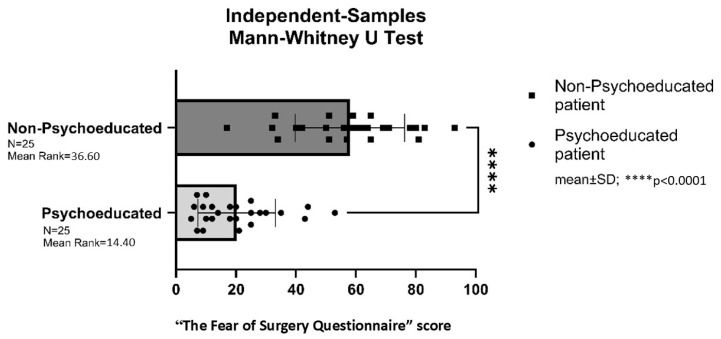
“The Fear of Surgery Questionnaire” score before surgery in the lumbar disc hernia (LDH) group. **** = *p* < 0.0001.

Patients in the psychoeducated LDH study group exhibited significantly lower preoperative anxiety levels compared to their non-psychoeducated counterparts in the LDH control group (*p* < 0.0001) (Figure 2).

### 3.2. Preoperative Anxiety in the PLIF Group

The “Surgical Fear Questionnaire” (SFQ) was completed by all patients in both the psychoeducated (PLIF study group) and non-psychoeducational (PLIF control group) groups prior to surgery.

**Figure 3:**
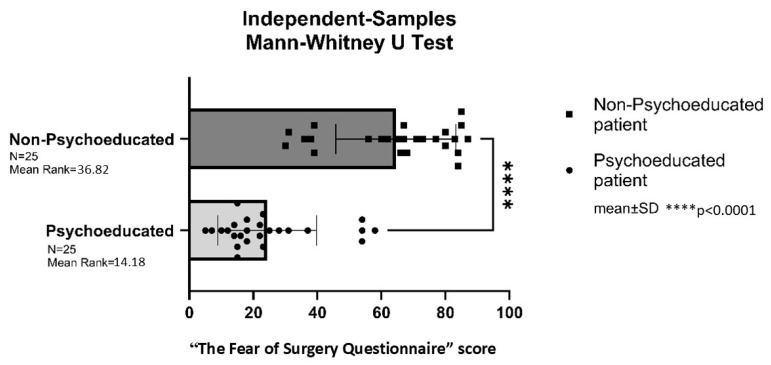
“The Fear of Surgery Questionnaire” score before surgery in the posterior lumbar interbody fusion (PLIF) group. **** = *p* < 0.0001.

Patients in the psychoeducated PLIF study group exhibited significantly lower preoperative anxiety levels compared to those in the non-psychoeducated PLIF control group (*p* < 0.0001) (Figure 3).

### 3.3. Postoperative Analgesic Consumption in the LDH Group

**Figure 4:**
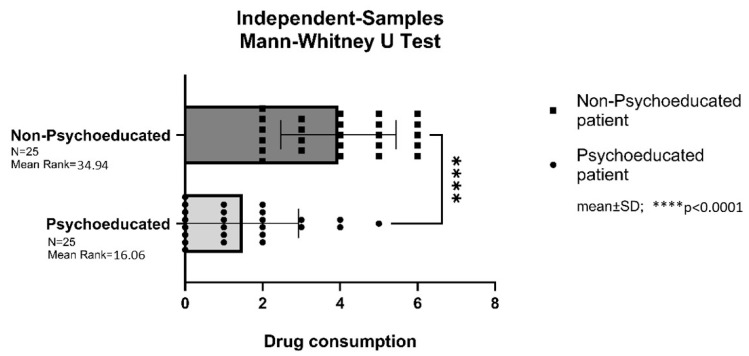
Postoperative analgesic consumption in the lumbar disc hernia (LDH) group. **** = *p* < 0.0001.

Patients in the non-psychoeducated LDH control group required significantly more pain medication after surgery compared to those in the psychoeducated LDH study group (*p* < 0.0001) (Figure 4).

### 3.4. Postoperative Analgesic Consumption in the PLIF Group

**Figure 5:**
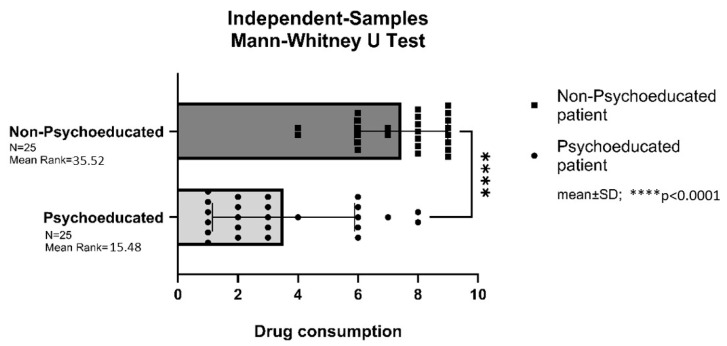
Postoperative analgesic consumption in the posterior lumbar interbody fusion (PLIF) group. **** = *p* < 0.0001.

Patients in the non-psychoeducated PLIF control group required significantly more pain medication after surgery compared to those in the psychoeducated PLIF study group (*p* < 0.0001) (Figure 5).

### 3.5. Correlation Between Anxiety and Analgesic Consumption in the LDH Group

A significant correlation was found between patients’ preoperative scores on the “Surgical Fear Questionnaire” and their postoperative analgesic consumption (*p* < 0.01). Specifically, the higher the preoperative anxiety score of LDH patients on the SFQ, the greater the postoperative drug demand (Table 2).

### 3.6. Correlation Between Anxiety and Analgesic Consumption in the PLIF Group

The results confirmed a significant correlation between the preoperative scores on the “Surgical Fear Questionnaire” and postoperative analgesic consumption in patients who received psychoeducation (PLIF study group) (*p* < 0.001). Specifically, the higher the preoperative anxiety score of spinal fusion patients on the SFQ, the greater the postoperative pain medication demand (Table 3).

### 3.7. Postoperative Mobilization in the PLIF Group

Patients in the psychoeducated PLIF study group were mobilized significantly earlier, on average one day earlier after surgery, compared to those in the non-psychoeducated PLIF control group (*p* < 0.0001).

**Figure 6:**
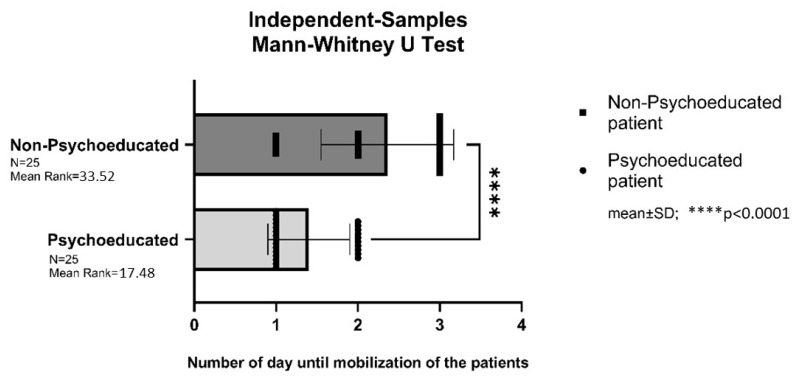
Postoperative mobilization in the PLIF group. **** = *p* < 0.0001.

The mobilization of patients in the lumbar herniated disc (LDH) group was not assessed, as all patients in this group were mobilized on the first day after surgery; therefore, the earlier mobilization was observed only in the PLIF group (Figure 6) (Table 4).

When interpreting the results of the present study, it should be taken into account that this was a single-center study and that the sample size of 25 patients per group necessitates further complementary investigations before clinical recommendations can be formulated. Furthermore, the lack of an attention-matched control, the allocation procedure, and the resulting limited generalizability should also be considered.

## 4. Discussion

### 4.1. Discussion and Conclusions

This study analyzed the effect of psychoeducation on postoperative analgesic consumption and mobilization following spine surgery. Patients undergoing lumbar disc herniation (LDH) surgery or monosegmental posterior lumbar intervertebral fusion (PLIF) were included in the analysis. Psychoeducated patients in both the LDH and PLIF groups showed significantly lower preoperative Surgical Fear Questionnaire (K score) values compared to non-psychoeducated controls. Postoperative drug consumption was significantly reduced in psychoeducated patients in both surgical groups, and lower preoperative K scores were associated with reduced postoperative analgesic demand. Earlier postoperative mobilization was observed only in the PLIF group, where psychoeducated patients were mobilized approximately one day earlier than controls.

Spine surgery poses significant challenges for patients, particularly in terms of perioperative pain management and postoperative mobilization. An epidemiological study found that 55.1% of patients with low back pain due to lumbar disc hernia sought medical care, and of these, 15.2% underwent surgery [11].

In our study, we hypothesized that psychoeducation would have a positive effect on patients undergoing spinal surgeries, potentially reducing postoperative analgesic consumption and mobilization time, thus promoting cost-effective patient care.

Preoperative anxiety has been shown to create a negative emotional state that hinders the patient’s recovery process [7,9]. Understanding the degree of anxiety helps to select more appropriate therapies to facilitate recovery. Fear of surgery is associated with increased postoperative pain, disability, and lower quality of life among patients undergoing various surgical procedures [16]. Engel et al. demonstrated that the higher the patient’s fear score, the poorer their preoperative psychological state, regardless of their diagnoses, medical history, or demographics [22]. In some related study, strong positive correlation was found between the quality of surgical nursing care and the knowledge provided to patients, indicating that patient education is linked to better nursing outcomes [23,24].

In summary, the literature suggests that psychoeducation contributes to the development of a postoperative sense of self-efficacy in patients, thereby facilitating a quicker return to a healthy lifestyle. Patients who receive psychoeducation often begin mobilization earlier and report better physical conditions compared to those who are forced to rest in bed due to pain following surgery [25,26,27,28]. It has also been described that when patients feel competent in their healing process, they experience less pain and become self-sufficient sooner. Therefore, it is in the best interest of surgeons to provide patients with a comprehensive therapy that follows a biopsychosocial approach, where psychoeducation is an integral component [16,29]. Psychological care can also reduce the clinic’s dependency on short-term painkillers, which are often used in large quantities [30,31].

In our study, the effect of preoperative psychoeducation was assessed in patients undergoing spine surgery. Psychoeducation was evaluated by measuring preoperative anxiety, postoperative analgesic consumption, and time to mobilization in patients who underwent either lumbar disc herniation surgery (hernio-discectomy, LDH) or spinal fusion for monosegmental lumbar instability (posterior lumbar interbody fusion, PLIF).

Our results demonstrated that psychoeducation had a beneficial effect on reducing patients’ preoperative fear of neurosurgical treatment, lowering the consumption of short-term pain-relieving medications, and decreasing the time to mobilization in PLIF patients. These findings are consistent with those of other studies that explored psychoeducation in different medical contexts [32,33,34]. As a result of the psychological care provided, the department experienced a decrease in short-term analgesic use, which in turn led to reduced healthcare costs associated with pain management [32,35,36,37,38]. Our findings confirmed the hypothesis that reducing preoperative anxiety through psychoeducation results in lower pain perception and earlier mobilization.

This study highlights the importance of integrating psychological care into surgical protocols. Our research confirmed our clinical experience; therefore, we consider it worthwhile to conduct similar studies in the future across multiple patient populations and with larger sample sizes. The presence of a psychologist in the surgical team can facilitate patient recovery by enhancing both physical and mental well-being, ultimately contributing to cost-effective patient care. This approach underscores the value of interdisciplinary collaboration, which can offer a pioneering model for modern surgical care.

### 4.2. Limitations

The limitations of the study include the difficulty of identifying a quiet, private setting suitable for individual psychoeducational sessions within a neurosurgical ward. An additional challenge is the availability of human resources, as neurosurgical departments may perform 5–10 spinal surgeries per day, rendering the provision of psychoeducation to patients a highly time-consuming process.

## Figures and Tables

**Table 1:** Clinical data of patients (LDH = monosegmental lumbar disc prolapse; PLIF = monosegmental posterior lumbar interbody fusion LIV = lumbal vertebra IV, LV = lumbal vertebra V, SI = sacral vertebra I).

	**LDH Study Group (N = 25)**	**LDH Control Group (N = 25)**
male (N)	11	12
female (N)	14	13
age (year, mean ± SD)	53.40 ± 11.14	52.88 ± 9.67
height (m, mean ± SD)	1.70 ± 0.10	1.68 ± 0.09
weight (kg, mean ± SD)	82.22 ± 12.65	79.52 ±11.67
BMI (mean ± SD)	28.37 ± 4.54	28.15 ± 4.40
LIV (level of surgery)	10	11
LV (level of surgery)	15	14
	**PLIF Study Group (N = 25)**	**PLIF Control Group (N = 25)**
male (N)	15	12
female (N)	10	13
age (year, mean ± SD)	58.12 ± 11.24	56.08 ± 8.87
height (m, mean ± SD)	1.71 ± 0.08	1.69 ± 0.09
weight (kg, mean ± SD)	81.04 ± 12.09	83.20 ± 13.65
BMI (mean ± SD)	27.66 ± 4.29	29.27 ± 5.23
LIV-V (level of surgery)	9	8
LV-SI (level of surgery)	16	17

**Table 2:** Correlation between anxiety and drug consumption in the LDH group. K = “Fear of surgery” questionnaire score. D = Drug consumption after spine surgery. N = number of cases. A significant correlation was found between patients’ preoperative scores on the “Surgical Fear Questionnaire” and their postoperative analgesic consumption. *** = *p* < 0.001.

Spearman’s rho	D	Correlation Coefficient	1.000	0.821 ***
		Sig. (2-tailed)	0.0	<0.001
		N	50	50
	K	Correlation Coefficient	0.821 ***	1.000
		Sig. (2-tailed)	<0.001	0.0
		N	50	50

**Table 3:** Correlation between anxiety and drug consumption in the psychoeducated PLIF group. K = “Fear of surgery” questionnaire score. D = Drug consumption after spine surgery. N = number of cases. The results confirmed a significant correlation between the preoperative scores on the “Surgical Fear Questionnaire” and postoperative analgesic consumption in patients who received psychoeducation (PLIF study group). *** = *p* < 0.001.

Spearman’s rho	D	Correlation Coefficient	1.000	0.922 ***
		Sig. (2-tailed)	0.0	<0.001
		N	25	25
	K	Correlation Coefficient	0.922 ***	1.000
		Sig. (2-tailed)	<0.001	0.0
		N	25	25

**Table 4:** “Fear of surgery” questionnaire score, drug consumption and postoperative mobilization. K = “Fear of surgery” questionnaire score. D = Drug consumption after spine surgery, M = number of days until postoperative mobilization. N = number of cases. The K-score was derived from the “Surgical Fear Questionnaire (SFQ)” completed by each patient prior to surgery. Medication usage was evaluated daily and categorized as follows: 0 = The patient required and received no additional analgesics beyond the standard prescribed routine for that day. 1 = The patient received additional metamizole sodium. 2 = The patient received additional diclofenac infusion. 3 = The patient received both metamizole sodium and diclofenac infusion. The drug score was evaluated daily throughout hospitalization and summed throughout hospitalization until discharge. The higher the “Fear of surgery” questionnaire score, the higher the drug consumption and the longer the duration until postoperative mobilization.

	**LDH Study Group (N = 25)**	**LDH Control Group (N = 25)**
K-score (mean ± SD)	20.24 ± 12.96	58.04 ± 18.27
D (day, mean ± SD)	1.48 ± 1.45	4.02 ± 1.51
	**PLIF Study Group (N = 25)**	**PLIF Control Group (N = 25)**
K-score (mean ± SD)	24.32 ± 15.42	64.52 ± 18.77
D (day, mean ± SD)	3.52 ± 2.37	7.44 ± 1.56
M (day, mean ± SD)	1.36 ± 0.49	2.36 ± 0.81

## Data Availability

Personal data of patients are stored safely, and not provided for any other third party. The data that support the findings of this study are available from the corresponding author upon reasonable request.

## Supplementary Materials

The following supporting information can be downloaded at: https://www.mdpi.com/article/10.3390/brainsci16020179/s1. File S1: Surgical Fear Questionnaire (SFQ).
