# Supplementary material for: Psychoeducation Reduces Postoperative Analgesic Consumption and Mobilization Period After Spine Surgery: A Controlled Clinical Trial
Source: Brain Sci. 2026 Jan 31;16(2):179. doi: 10.3390/brainsci16020179 (PMC12939080; doi:10.3390/brainsci16020179)
Supplement: Supplementary file 1 [file brainsci-16-00179-s001.zip › brainsci-4083239-supplementary.pdf]

Supplementary Materials:

Surgical Fear Questionnaire

With the help of the questionnaire, we would like to assess your fears regarding the surgical procedure you undergo.

Please circle the number that you think the best reflects your current feelings.

1. I'm afraid of the surgery.

0 1 2 3 4 5 6 7 8 9 10

I'm not afraid at all. I'm really afraid.

2. I'm afraid of anesthesia.

0 1 2 3 4 5 6 7 8 9 10

I'm not afraid at all. I'm really afraid.

3. I am afraid of the pain after surgery.

0 1 2 3 4 5 6 7 8 9 10

I'm not afraid at all. I'm really afraid.

4. I am afraid of unpleasant side effects after surgery (such as nausea).

0 1 2 3 4 5 6 7 8 9 10

I'm not afraid at all. I'm really afraid.

5. I am afraid that my health will deteriorate because of the operation.

0 1 2 3 4 5 6 7 8 9 10

I'm not afraid at all. I'm really afraid.

6. I'm afraid the surgery fails.

0 1 2 3 4 5 6 7 8 9 10

I'm not afraid at all. I'm really afraid.

7. I'm afraid of hospital staying.

0 1 2 3 4 5 6 7 8 9 10

I'm not afraid at all. I'm really afraid.

8. I'm afraid of my family.

0 1 2 3 4 5 6 7 8 9 10

I'm not afraid at all. I'm really afraid.

9. I'm afraid I won't fully recover from the surgery.

0 1 2 3 4 5 6 7 8 9 10

I'm not afraid at all. I'm really afraid.

10. I am afraid of the long rehabilitation after surgery.

0 1 2 3 4 5 6 7 8 9 10

I'm not afraid at all. I'm really afraid.
